# Design of a high-mobility multi-terrain robot based on eccentric paddle mechanism

**DOI:** 10.1186/s40638-016-0041-3

**Published:** 2016-06-07

**Authors:** Yi Sun, Yang Yang, Shugen Ma, Huayan Pu

**Affiliations:** Department of Robotics, Ristumeikan University, 1-1-1 Noji Higashi, Kusatsu, Shiga 525-8577 Japan; Department of Mechanical and Aerospace Systems Engineering, Tokyo Institute of Technology, Ookayama, Megoru-ku, Tokyo, 152-8552 Japan; School of Mechatronic Engineering and Automation, Shanghai University, 149 Yanchang Road, Shanghai, China

**Keywords:** Eccentric paddle mechanism, ePaddle, Multi-terrain locomotion

## Abstract

Gaining high mobility on versatile terrains is a crucial target for designing a mobile robot toward tasks such as search and rescue, scientific exploration, and environment monitoring. Inspired by dextrous limb motion of animals, a novel form of locomotion has been established in our previous study, by proposing an eccentric paddle mechanism (ePaddle) for integrating paddling motion into a traditional wheeled mechanism. In this paper, prototypes of an ePaddle mechanism and an ePaddle-based quadruped robot are presented. Several locomotion modes, including wheeled rolling, legged crawling, legged race-walking, rotational paddling, oscillating paddling, and paddle-aided rolling, are experimentally verified on testbeds with fabricated prototypes. Experimental results confirm that paddle’s motion is useful in all the locomotion modes.

## Background

As learned from realistic search and rescue activities at disaster sites, the mobility of the mobile robot is of the greatest importance [1]. To guarantee its mobility, the locomotion mechanism of the robot should be able to adapt to diverse terrestrial, aquatic, and amphibious terrains. Robots based on single locomotive forms lack such crucial multi-terrain adaptivity which prevents them from being practically used in realistic search and rescue activities.

Recently, hybrid locomotion mechanisms have been proposed toward multi-terrain mobility for mobile robot. Most of them are designated for terrestrial-task-oriented robots, which consist of the following two major groups. The first group includes those robots that are composites by basic motion units such as wheels and legs or those units that act together to provide locomotion. Most of them are articulated-wheeled robots which have wheels placed at the end of the legs, including Shrimp [2], Octopus [3], ATHLETE [4], Hylos [5], Roller-Walker [6], AZIMUT [7] and Robot IMR-Type I [8]. Others have separated legs and wheels that can behave as a legged robot and a wheeled robot cooperatively and simultaneously. For instance, Chariot-II constructed at Tohoku University (Japan) has two big active wheels at the both sides of the body and four legs with three joints at the both ends of the body [9]. ALDURO proposed by Hiller et al. [10] has wheels on the back legs but no wheels on the front legs. Wheeleg [11] has two front legs, each with three-DOF prismatic joints, and two independently actuated back wheels.

Robots in the second group have kinematically synthesized locomotion mechanisms which are multi-functional. For example, Hong et al. proposed a mechanism consisting of two rimless wheels with individually actuated spokes in Intelligent Mobility Platform with Active Spoke System (IMPASS) to travel on uneven surfaces like tracks and step over obstacles like legged vehicles while retaining the simplicity of wheels [12]. Perpendicularly Oriented Planetary LEgged Robot (PEOPLER-II) proposed by Okada et al. [13] uses pairs of legs jointed at a wheel rim so that the leg can fold in walking and unfold in rolling. Steffan et al. presented the idea of cellular locomotion mechanism in [14], where the proposed mechanism generates rectilinear locomotion exclusively through linear actuation of the legs. In [15], the authors discussed a rimless wheel with radially expanding legs, intending to travel over obstacles by expanding the wheel instead of avoiding obstacles. A bipedal robot that combines the rolling, walking, and climbing locomotion was proposed by Shores et al. [16]. Lin et al. proposed a wheel-leg hybrid robot that can change the morphology of full-circle wheels into half-circle legs using a transformation mechanism [17, 18]. To change locomotion mode, robots in this category usually need performing shape alternating procedures.

Several amphibious robots are based on wheels [19] and tracks [20]. Some robots are inspired from natural creatures, such as robot turtle by Low et al. [21], crab-like robot by Wang et al. [22], lobster by Ayers et al. [23], and snake-like robot AmphiBot I and II by Crespi et al. [24, 25]. We also have developed an amphibious snake-like robot [26]. Other robots with novel hybrid mechanisms have been reported as well. For instance, an autonomous amphibious vehicle which can travel on aquatic and terrestrial terrains by using four driven paddle wheels was presented by Frejek et al. [27]. Dubbed Whegs™ IV [28], which is based on Whegs™, has six wheel legs and is capable of walking in a tripod legged gait and swimming underwater.

However, limited by their locomotion ability, above amphibious robots still cannot be applied for search and rescue tasks after tsunami, where multiple gaits on terrestrial, aquatic, and amphibious terrains are required.

**Fig. 1:**
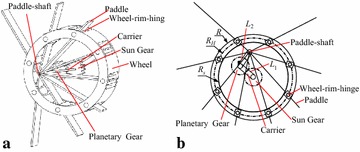
Concept of the eccentric paddle mechanism. **a** Major components in an ePaddle module. **b** Schematic drawing of the ePaddle mechanism

**Fig. 2:**
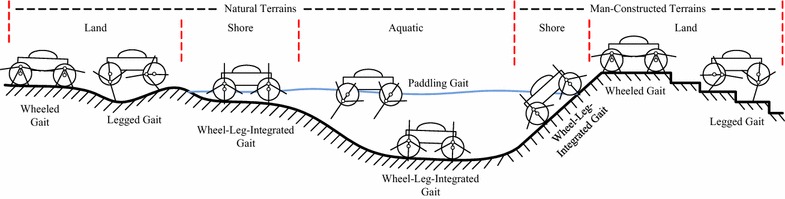
Multi-terrain locomotion modes. An ePaddle-based robot is capable of performing versatile gaits in complex environments [30]

We start our effort to develop an amphibious robot that possesses high mobility and high energetic efficiency in complex amphibious environment by proposing a novel locomotion mechanism: eccentric paddle mechanism (ePaddle) [29]. The concept of the ePaddle is shown in Fig. 1. By actively locating the paddle-shaft inside the wheeled shell via independent actuators, motion patterns of the ePaddle can be alternated. Five locomotion gaits (as shown in Fig. 2) have been proposed in our previous work, three of which are terrestrial gaits, namely the wheeled rolling gait [30], the legged walking gait [31, 32], and the legged-wheeled hybrid gait [33, 34]; and two of which are aquatic gaits, named the rotational paddling gait [35] and the oscillating paddling gait [36, 37]. The ability to generate thrusts for underwater propulsion has been observed as well [36, 37].

Based on our preliminary analysis or experiments on locomotion modes of an ePaddle mechanism, interesting findings relative to how paddle’s motion could improve environmental adaptability have been noticed. In this paper, the effects of paddle’s motion on multi-terrain locomotion are focused on. Prototypes of the ePaddle module and an ePaddle-based quadruped robot are fabricated for experimental verification. Experimental results show that paddle-aided motion is useful for generating vectored thrusts in aquatic environment, for improving soil reaction forces on soft sandy terrain, and for enhancing obstacle-negotiating capability on rough terrains.

## Methods

### Concept of the eccentric paddle mechanism

Legged locomotion modes, such as running, climbing, and paddling, bring remarkable multi-terrain adaptivity to animals. However, wheeled motion is superior to legged one in speed and energetic efficiency. The best way to acquire multi-terrain adaptivity, speed, and energetic efficiency for an mobile robot is finding a method to integrate legged motion into wheeled locomotion.

Based on this idea, concept of the eccentric paddle mechanism (ePaddle), as shown in Fig. 1, has been proposed firstly in [30]. The ePaddle mechanism consists of a wheeled shell, a set of rigid paddles, a paddle-shaft, paddle-hinges, and wheel-rim-hinges.

A wheeled shell of the ePaddle module is actuated by a motor to rotate around the wheel axis, and a set of paddles are mounted on and can passively rotate around a paddle-shaft inside the wheel. Wheel-rim-hinges placed on the wheel’s rim hold the paddles and allow the paddles to passively slide through them. Location of the paddle-shaft with respect to the wheel axis is actively positioned by two motors via a planetary gear train.

By tuning paddles’ motion, versatility in locomotion modes is brought to the ePaddle mechanism. Figure 2 demonstrates several examples of paddle-aided motions that are feasible for an ePaddle-based robot.

### Design of the ePaddle-based prototype robot

#### Prototype ePaddle module

**Fig. 3:**
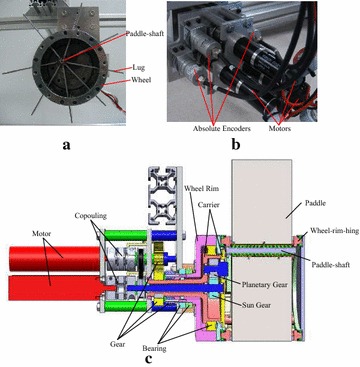
Prototype design of the ePaddle module. **a** Front view and **b** rear view of an assembled prototype module. **c** Section view of the CAD drawing of the module

The fabricated ePaddle prototype module is shown in Fig. 3a. Key components, such as the wheel-rim-hinge, the paddle-hinge, and the transmission chains of the prototype module, are shown in Fig. 3b, c.

As shown in Fig. 3c, the wheel rim, carrier, and sun gear are separately driven by three DC brushed motors [Maxon RE25, integrated with a GP gearbox (72:1)] via gears. The angular positions of the three joints are collected by three absolute encoders (RE22, RLS; Slovenia). The wheel rim is rotated by the first motor through a pair of flat gears. The position of the paddle-shaft is determined by the angular position of the carrier and sun gear of planetary gear mechanism.

The stainless steel paddle-shaft is fixed to the disk integrated with the planetary gear by pins and bolts. As shown in Fig. 3c, the paddles are made from rectangular rigid aluminum plates, and each paddle is hung on the paddle-shaft by a paddle-hinge. Ball bearings are assembled in the paddle-hinge, ensuring smooth passive rotation of the paddle around the paddle-shaft.

The wheeled shell, as shown in Fig. 3c, is constructed from two annulus frames, eight shell pieces, and eight wheel-rim-hinges. The shells are fixed between the frames by stainless steel pins and bolts to form the wheel surface. The wheel-rim-hinges, which freely rotate around their central axis, are supported between the frames by the bearings. The paddle slides through a hole inserted in the wheel-rim-hinge.

For dust-proofing, the clearances between the paddle and the wheel-rim-hinge and between the wheel-rim-hinge and the shell are filled with woolen felt sheets. The outer surface of the wheel is polished for future adhesion of rubber tire.

Specifications of the ePaddle module are listed in Table 1.

**Table 1:** Specifications of the ePaddle prototype module

Item	Description
Paddle	
Dimensions	Length, *L*: 96.5 mm; width: 63 mm; thickness: 2 mm
Moving range	$R_S = L_A + L_B = 40$ mm, where $L_A = L_B$
Material	Aluminum alloy (A5052)
Weight	36 g (eight lugs with eight paddle-hinges)
Wheel shell	
Dimensions	Outer radius, $R_A$: 56.5 mm; width: 113 mm;
Material	Aluminum alloy (A5052)
Wheel-rim-hinges	
Dimensions	Pitch circle radius, $R_H$: 50 mm
Materials	Aluminum alloy (A5052)
Assembled module	
Weight	3.7 kg (with three motors)

#### Kinematics of the ePaddle mechanism

**Fig. 4:**
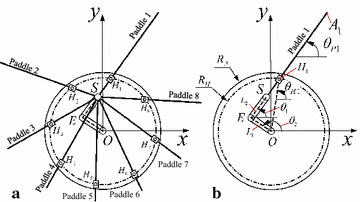
Kinematics of the ePaddle module. **a** Coordinates definitions. **b** Definitions of the joint angles (only one of the paddle is shown for better clarity)

Kinematics of the ePaddle mechanism describes kinematic relationship between the lug’s motion and joint’s motion. As shown in Fig. 4a, a coordinate system named wheel coordinates is defined in the way that the origins of the coordinates locate at the wheel center, and the *x*-axis is in the horizontal direction. Posture of the ePaddle, or in other word, positions and orientations of ePaddle’s components with respect to the wheel coordinates, is determined by angular positions of the wheel joint, the base joint, and the arm joint, respectively, denoted by three joint parameters, $\theta _0$, $\theta _1$, and $\theta _2$.

For example, as shown in Fig. 4b, inclination angle and tip position of paddle-1 can be, respectively, expressed by

1 $${\theta _{Pi}} = {\text {atan }} 2({y_{Hi}} - {y_S},{x_{Hi}} - {x_S})$$

and

2 $$\begin{aligned} \left\{ \begin{array}{ll} {x_{Pi}} = {x_S} + L\cos {\theta _{Pi}} \\ {y_{Pi}} = {y_S} + L\sin {\theta _{Pi}} \\ \end{array}\right. \end{aligned}$$

where ${S} = {[{x_S},{y_S}]^T}$ and ${H}_{i} = {[{x_{Hi}},{y_{Hi}}]^T}$ are the position of the paddle-shaft and the position of the wheel-rim-hinge-1, respectively, and *L* is the length of the paddle. They can be expressed by

3 $$\begin{aligned} \left\{ {\begin{array}{*{20}{c}} {{x_S} = {L_1}\cos {\theta _1} + {L_2}\cos {\theta _2}} \\ {{y_S} = {L_1}\sin {\theta _1} + {L_2}\sin {\theta _2}} \end{array}} \right. \end{aligned}$$

and

4 $$\begin{aligned} \left\{ {\begin{array}{*{20}{c}} {{x_{Hi}} = {R_H}\cos {\theta _{Hi}}} \\ {{y_{Hi}} = {R_H}\sin {\theta _{Hi}}} \end{array}} \right. \end{aligned}$$

where ${\theta _{Hi}} = {\theta _0} + (i - 1)\pi /2$ is the posture angle of the wheel-rim-hinge-*i*; ${L_1}$ and ${L_2}$ are lengths of the base link and the arm link, respectively; ${R_H}$ is the radius of the circular where the wheel-rim-hinges locate.

#### ePaddle-based quadruped robot

**Fig. 5:**
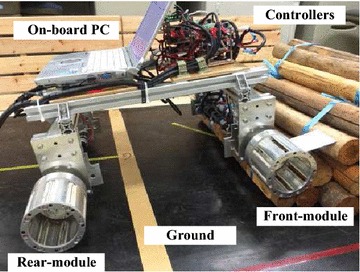
Prototype of an ePaddle-based quadruped robot. Four ePaddle modules are mounted on the robot body where a network of custom-designed real-time controllers and an onboard computer are installed. The robot can be powered by either a power tether or a onboard battery

An ePaddle-based quadruped robot equipped with four ePaddle modules has been assembled as shown in Fig. 5. A controller network consisting of multiple motor controllers and a commander computer is carried on body of the robot. A power tether is used for supplying power source to the robot for conducting indoor experiments, whereas an onboard battery is used for outdoor experiments.

**Fig. 6:**
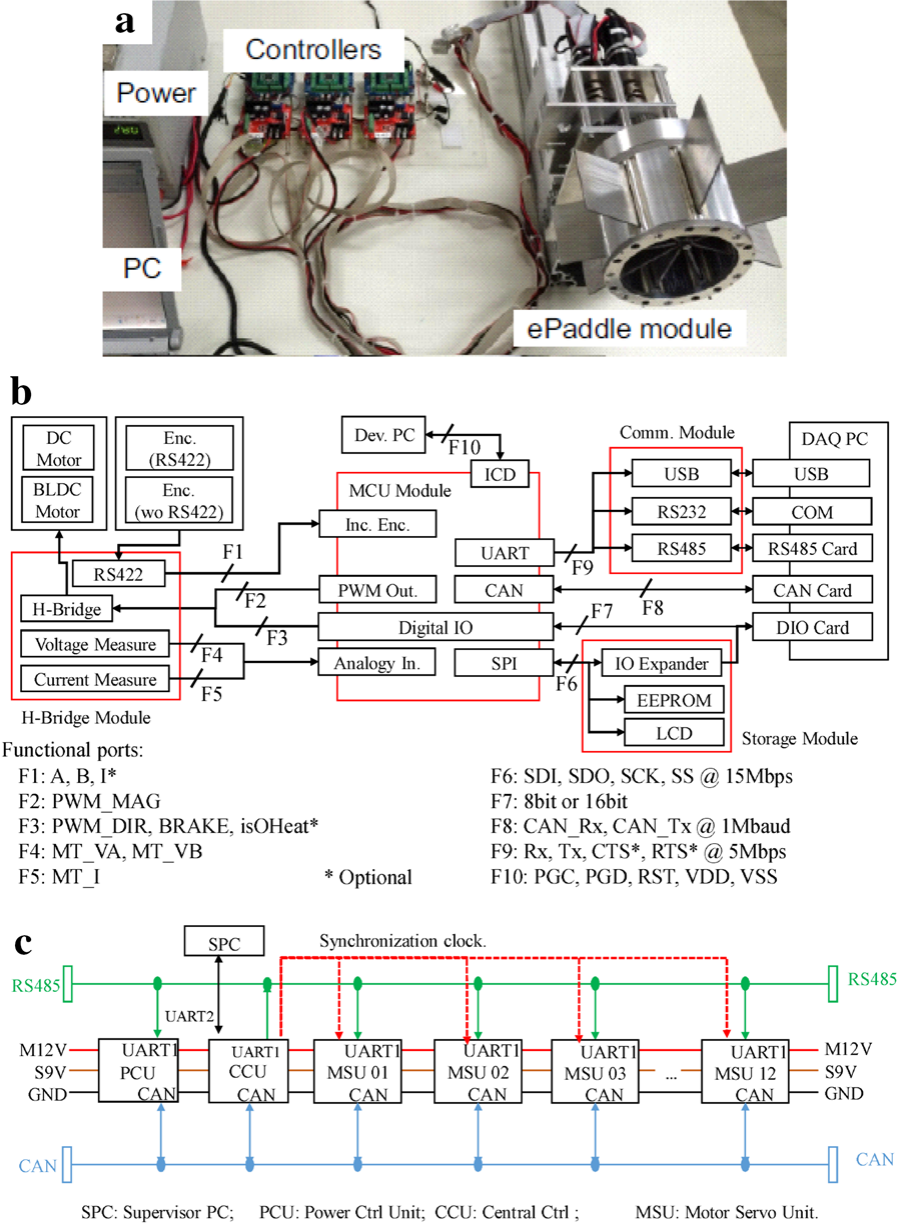
Custom-designed reconfigurable motion controller. **a** Prototype. **b** Schematic drawing of the controller. **c** Schematic drawing of the controller network

The real-time motion controller, as shown in Fig. 6a, based on a dsPIC33-FJ128MC804 microcontroller (Microchip, USA) is custom-designed in a highly reconfigurable way to enable easy reconfiguration of the controller network without major modifications in hardware. The schematic drawings of the motion controller and the controller network are shown in Fig. 6a, b, respectively. Figure 6c shows an assembled controller network for an ePaddle module.

### Multi-terrain locomotion modes

At current stage, five types of feasible locomotion modes have been found for an ePaddle-based robot, which are summarized in Fig. 2. In addition, a novel hybrid locomotion mode named Wheel-leg-integrated Mode (or Active Lugged Mode) is found to be helpful when the robot travels on soft terrains.

#### Wheeled rolling mode

A wheeled rolling motion can be performed by making the outer surfaces of the shells in contact with ground and rolling as wheels. The paddle-shaft is located at a proper position to pretend the paddles punching with the ground.

To avoid paddle-ground-collision two critical requirements should be fulfilled in designing the ePaddle module and planning its wheeled motion. The first one is that the shortest protruding length of the paddles should be zero, which means the following geometric constraint should be satisfied:

5 $$\begin{aligned} L \le R_{S} + R_A \end{aligned}$$

where $R_S = \sqrt{(}x^2_S + y^2_{S})$ is the radius of the workspace of paddle-shaft and $R_A$ is the radius of the wheel. In our prototype, we let $L = R_S + R_A$ for maximizing the workspace of the paddles.

**Fig. 7:**
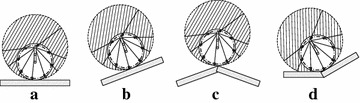
Locating paddle-shaft at a proper place for avoiding paddle-ground collision in wheeled mode [30]. **a** On even ground, **b** on a slope, **c** on the top of a slope, **d** on the bottom of a slope

The second requirement is that the paddle-shaft should be located at a proper position for ensuring the landing paddle is totally retracted when the landing paddle touches the ground. As presented in [30], a practical choice of paddle-shaft’s position is the farthest position away from the contact point. Figure 7 shows several examples of selecting positions of paddle-shaft on variety of terrains.

The advantage of the wheeled rolling mode is its simplicity in control. Once the paddle-shaft is properly located, only the wheel is actively actuated. Comparing with traditional wheeled rolling, the presence of the paddles improves obstacle-negotiating capability of the ePaddle module by allowing the module to “hook” or “climb” on the obstacles.

#### Aquatic paddling modes

In an aquatic environment, two types of swimming motions, namely the rotational paddling and the oscillating paddling, can be performed by an ePaddle mechanism.

**Fig. 8:**
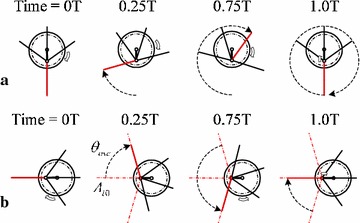
Two feasible paddling modes of the ePaddle mechanism. **a** Rotational paddling motion. **b** Oscillating paddling motion

The first type is the *rotational paddling motion* [35], as shown in Fig. 8a. During this gait, all the paddles are driven by the wheel to rotate in one direction around the paddle-shaft. Because the paddle-shaft is located eccentrically, the protruding areas of the paddles are different, which generate different amount of thrusts. By alternating the location of the paddle-shaft and rotational speed of the wheel, we can change the magnitude and direction of the thrusts.

The second aquatic motion, as shown in Fig. 8b, is the *oscillating paddling motion* [38]. For our ePaddle mechanism, we initially choose one of the four paddles in the ePaddle as the working paddle. The paddle-shaft is located at a position with an eccentric distance $r_S$. The wheel-like shell is driven to rotate, but the rotational direction is periodically reversed. It will result in the oscillation of the working paddle at an average position $A_{i0}$ with an angular magnitude $\theta _{\rm Osc}$. The direction and magnitude of the generated thrusts are adjustable by changing $A_{i0}$ and $\theta _{\rm Osc}$, respectively.

To generate smooth oscillating motion, the angular position of the wheel is driven to follow sinusoidal-based trajectories defined as

6 $$\begin{aligned} \theta _{W, {\rm sym}} = \theta _{\rm Osc}\sin \left( \frac{2\pi {t}}{T}+\frac{\pi }{2}\right) . \end{aligned}$$

#### Legged walking modes

The paddles integrated in the ePaddle mechanism also have the ability to serve as legs on rough terrains in two different modes.

The first type is the *crawling motion mode* that is similar to the crawling gait of traditional open-link based legged robots [39]. For a ePaddle-based quadruped robot working in this mode, three of the four legs are in contact with the ground to support the weight of the robot, and another one leg is swinging in the air. Detailed discussion on this mode has been presented in [30].

The second legged mode is the novel *race-walking motion mode*, which is a unique legged motion for the ePaddle mechanism. For an ePaddle-based quadruped robot working in this mode, all the four ePaddle modules are always in contact with the ground during the locomotion which results the robot is supported by at least four legs at any time. Planning method of this mode has been discussed in [31].

#### Active-lugged mode (Wheel-leg-integraged mode)

On sandy or muddy soft terrains, both legs and wheels may fail due to the limited contact areas and the sliding, respectively. An ePaddle-based robot can perform *active-lugged locomotion mode* by digging its paddles into the soil to generate additional supporting or tracking forces.

**Fig. 9:**
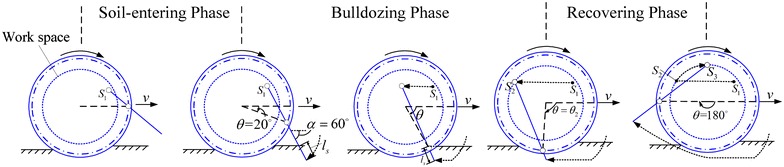
Standard sequence of the active-lugged locomotion

Standard sequence of the active-lugged locomotion, as shown in Fig. 9, consists of three phases: the soil-entering, bulldozing, and recovering phase, according to paddle’s motion. In all the phases, the wheel is driven to rotate at a constant speed, whereas the paddle-shaft is actively actuated to follow a predefined trajectory. Paddle-shaft’s trajectory is designed in a way that a desired planar motion of the paddle can be got in the bulldozing phase. Pure translational and pure rotational motions are two of the example planar motions of the paddle in the bulldozing phase.

### Setups of locomotion experiments

#### Aquatic paddling experiments

**Fig. 10:**
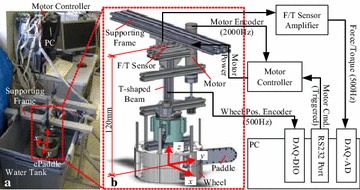
Testbed with a water tank for measuring aquatic thrust forces. **a** Assembled water tank. **b** Schematic drawing of the data acquisition system

As shown in Fig. 10, a simplified ePaddle module equipped with four paddles was mounted on an aluminum frame and submerged beneath the water surface of a water tank. A force/torque sensor holding the aluminum frame was fixed on the supporting frame above the water surface for measuring the thrust forces generated by the ePaddle module. Sampling rate of the measurement system was 1000 Hz.

#### Sandy rolling experiments

**Fig. 11:**
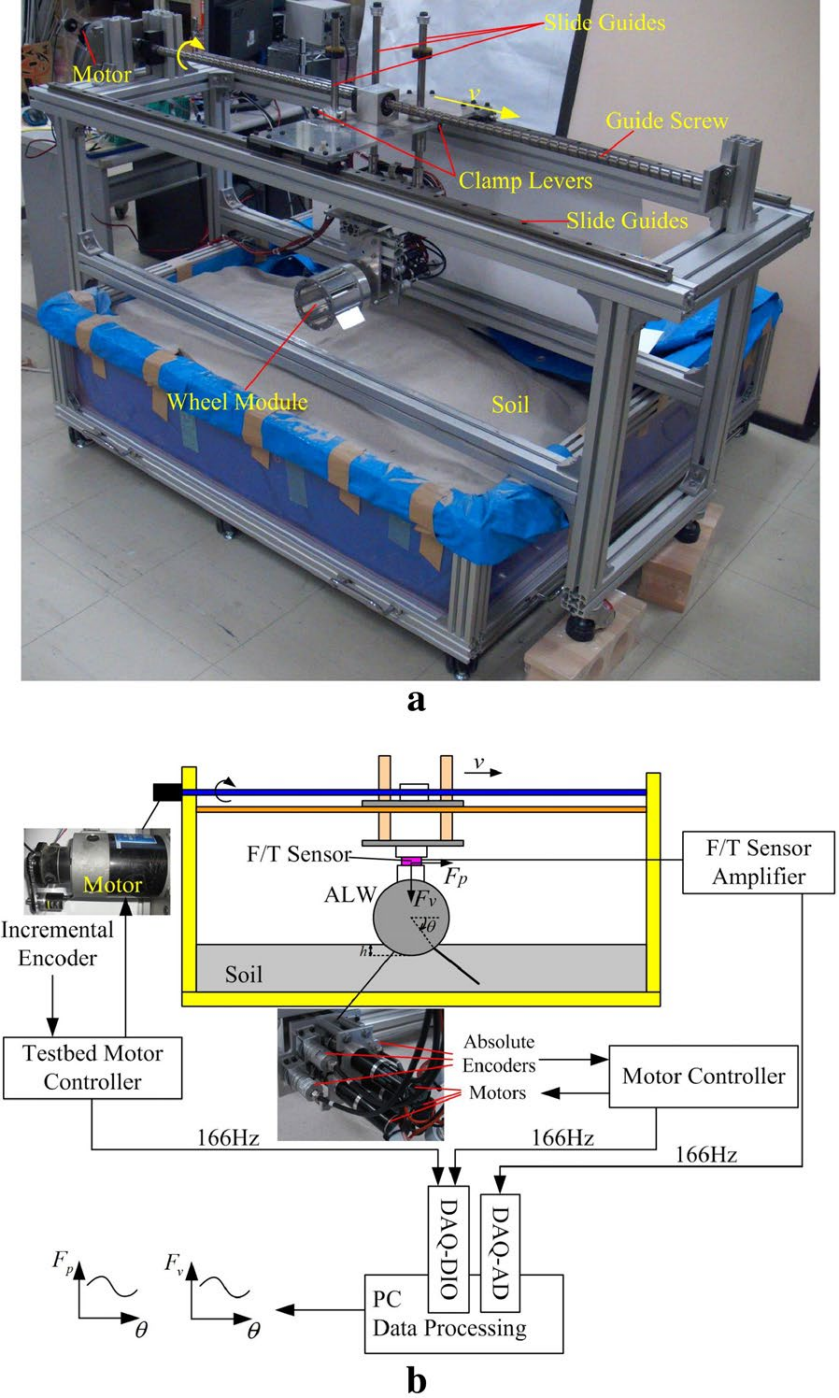
Sandy testbed for measuring soil-ePaddle reaction mechanics. **a** Assembled testbed. **b** Schematic drawing of the data acquisition system

A testbed of length 1700 mm, width 500 mm, and height 800 mm was designed for conducting locomotion experiments on sandy terrain, as shown in Fig. 11. A linear conveyance unit was actuated at desired speeds by a DC brushed servo motor via a ball-bearing screw. A fully functional ePaddle module was mounted on the conveyance unit via a six-axis force/torque sensor (Delta SI-330-30, ATI; USA). Vertical position of the ePaddle module with respect to the ground surface was fixed during the experiments for eliminating influence of wheel’s sinkage on measured soil reaction forces.

Angular position of the ball-bearing screw and angular positions of the three joints of the ePaddle module were, respectively, measured by an incremental encoder (E6A2-CWZ3E, OMRON; Japan) and three absolute encoders (RE22, RLS, Slovenia), both of which were sampled at 166 Hz through a digital input/output board (NI-6001, National Instrument; USA) on a PC running a Windows XP operating system. The force signals were sampled by an A/D board (AD12-16(PCI), Contec; Japan) at the same rate as the encoders.

Soft dry sand filled in the sandbox of the testbed has been purified, sieved, ventilated, and dried. The physical and mechanical properties of the soil are listed in Table 2.

**Table 2:** Soil parameters

Parameters	Unit	Value
Cohesion stress *c*	Pa	400
Soil friction angle $\phi$	deg	38.1
Soil specific weight $\gamma$	$\hbox {kg/m}^3$	1480
Adhesion stress $c_a$	Pa	66
Lug–soil friction angle $\delta$	deg	10.4

#### Terrestrial walking experiments

A single ePaddle prototype module was tested in several scenarios to verify the terrestrial locomotion. The tested scenarios include rolling on even ground, climbing up wood steps, ascending a wood slope of several inclination angles, and negotiating with stacks of wood poles.

A quadruped prototype robot equipped with four ePaddle modules, as shown in Fig. 5, has been fabricated to verify the proposed controller network as well.

## Results and discussion

### Generating vectored thrusts in aquatic environment

**Fig. 12:**
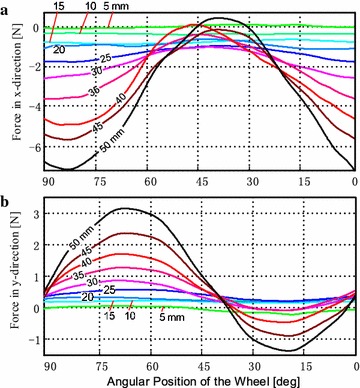
Thrusts generated in rotational paddling mode. Measured thrusts in **a** *x*-direction and in **b** *y*-direction with eccentric distances between the paddle-shaft and the wheel center varying from 0 to 50 mm and the wheel rotates at 50 rpm along the direction. The *number on the line* indicates the eccentric distance in millimeter (mm)

Figure 12 shows the experimental results of the rotational paddling modes with the paddle-shaft locating at an eccentric position along the *y*-axis. Several distances between the paddle-shaft and the wheel center were used in the experiments.

Experimental results in Fig. 12 confirm that asymmetric thrusts can be generated in the *x*-direction by placing the paddle-shaft at an eccentric position with respect to wheel center along the *y*-direction. It implies that vectored thrusts are achievable by tuning paddle-shaft’s position.

**Fig. 13:**
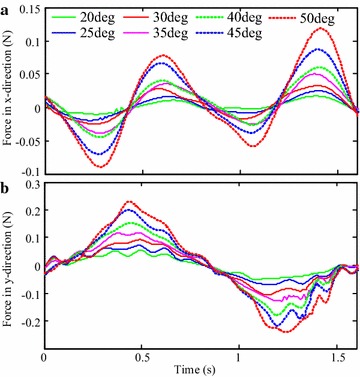
Thrusts generated in oscillating paddling mode. Measured thrusts in **a** *x*-direction and in **b** *y*-direction with different oscillation amplitudes. The oscillation period is 1.5 s and the paddle-shaft is placed at its outmost position along the *x*-axis

Figure 13 shows measured thrusts for oscillating paddling experiments with various oscillation amplitudes. As shown in the figure, thrusts in *x*-direction have asymmetric magnitudes, whereas thrusts in *y*-direction follow a symmetric way. Based on this result, the capability of generating vectored trusts of the ePaddle module in the oscillating mode is experimentally confirmed as well.

### ePaddle-soil reaction mechanics in sandy terrain

**Fig. 14:**
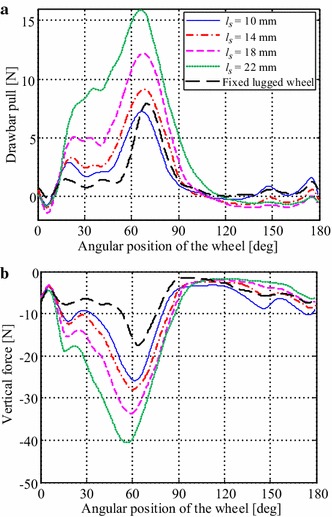
Soil-ePaddle interaction mechanics. Comparison of **a** drawbar pulls and **b** vertical forces generated by a fixed lugged wheel (lug height = 13 mm) and by the ePaddle mechanism at various of sinkage lengths and 60° inclination angle

As shown in Fig. 14, the drawbar pull and vertical force of the ePaddle module were measured and compared with the results of a fixed lugged wheel that has the same radius as the ePaddle’s wheel.

Results in Fig. 14 reveal the following advantages of the ePaddle module rolling on sandy terrain. The ePaddle mechanism is able to insert the paddle into the soil earlier in the cycle and depart from the soil later to increase the drawbar pull and vertical force over a wider range. Compared with the fixed lugged wheel with a maximum sinkage length of 18 mm, the maximum drawbar pull of the ePaddle module at 18 mm sinkage length is increased by 53 %. In addition, the ePaddle mechanism is able to significantly enhance the vertical reaction force on sandy soil.

### Obstacle-negotiating capability

**Fig. 15:**
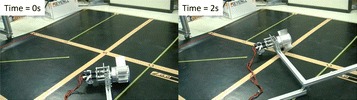
Rolling of an ePaddle module on even ground

Experimental results of wheeled rolling on even ground, as shown in Fig. 15, confirmed that by locating the paddle-shaft at a proper position, the paddle-ground collision can be completely avoided.

**Fig. 16:**
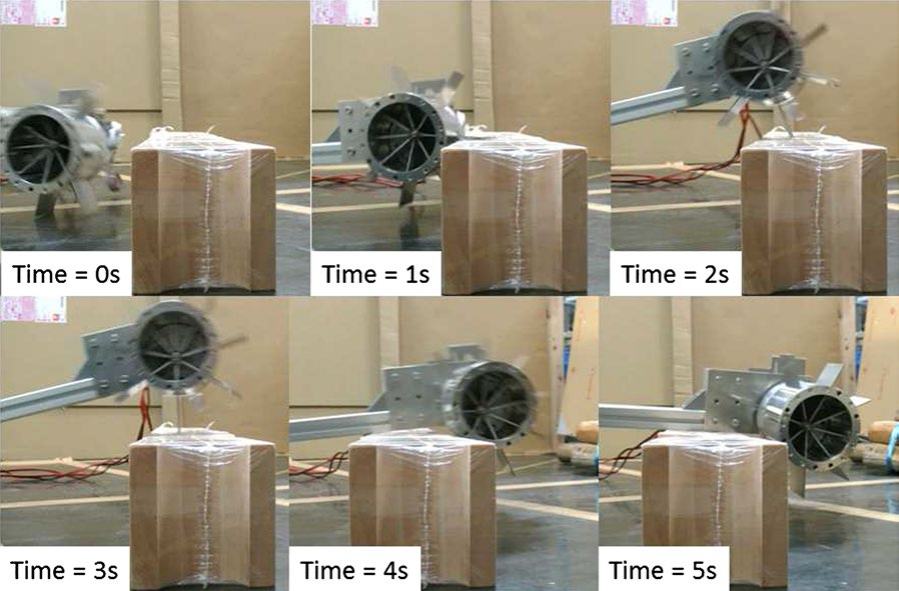
Results of step-climbing experiment. An ePaddle module successfully climbed a 100 mm height wood block

Wood blocks of 100 mm height (which is about 1.78 times of wheel radius) were placed on the ground to test the step-climbing capability of the ePaddle module. Experimental results, as shown in Fig. 16, verified that the ePaddle mechanism has great advantage on climbing high obstacle thanks to the paddle’s motion.

**Fig. 17:**
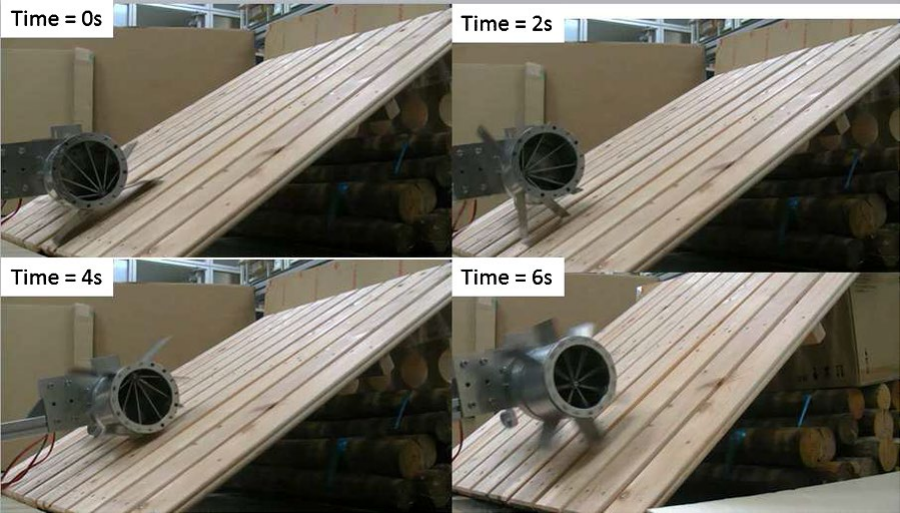
Results of the climbing experiments on a slope of 45°

Experimental results, as shown in Fig. 17, confirmed that the ePaddle module is able to climb a slope of up to 45°.

**Fig. 18:**
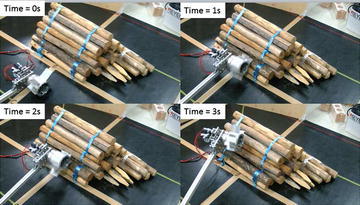
The ePaddle module is climbing stacks of wood poles

Mobility on rough terrains is tested with stacks of wood poles with heights of 210 and 330 mm as shown in Fig. 18. The paddle can easily firmly hook on the poles due to their rugged shape, which suggested that the ePaddle module has great obstacle-negotiating capability when accessing rugged obstacles.

**Fig. 19:**
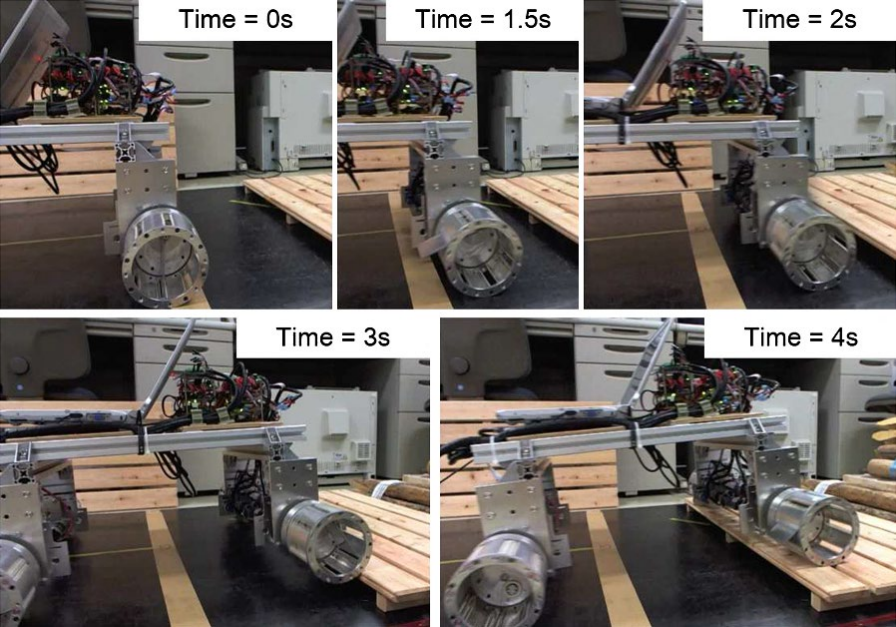
Experiment result of the paddle-aided stair climbing. The paddle-shaft is placed at its upmost position inside the wheel. Paddle’s motion enables the robot to climb the step

The prototype robot, as shown in Fig. 5, was used to test the step-climbing capability of an ePaddle-based quadruped robot. Results shown in Fig. 19 confirmed that the robot is able to climb the stair due to the paddle–stair interaction. This result confirmed that the paddle’s motion is useful during stair climbing, even the stair is slippy to wheels.

## Conclusions

In this paper, prototype designs of an ePaddle mechanism and an ePaddle-based quadruped robot have been proposed. By integrating paddles into a traditional wheel mechanism, the ePaddle mechanism is superior to other locomotion mechanisms due to its advantage in locomotion versatility and terrain adaptability.

Principles of multi-terrain locomotion modes of the ePaddle mechanism have been discussed and experimentally verified with the prototypes on fabricated testbeds.

Experimental results have confirmed that paddle’s motion, which is actively controlled by locating the paddle-shaft, is useful in all the feasible locomotion modes. For example, paddles can generate vectored thrusts in aquatic environments with two paddling modes. On sandy terrains, paddle’s motion can generate larger drawbar pull and vertical forces than traditional fixed-lug wheel. In the wheeled rolling mode, paddles can help the robot to climb high obstacles.

In our future study, coordinating movements among the four ePaddle modules in the prototype robot will be considered to further improve the mobility on rough terrains. Optimization of the locomotion modes taking the energetic efficiency into account will be investigated as well.

## References

[1] Murphy RR. An overview of robots from USA at Fukushima and the tsunami recovery. In: Special forum report at 2011 IEEE international conference on robotics and automation (ICRA’11). Shanghai, China 2011

[2] Estier T, Crausaz Y, Merminod B, Lauria M, Piguet RRS. An innovative space rover with extended climbing abilities. In: Proceedings of space and robotics 2000. Albuquerque, USA, 2000

[3] Lauria M, Piguet Y, Siegwart R. Octopus—an autonomous wheeled climbing robot. In: Proceedings of the fifth international conference on climbing and walking robots (CLAWAR’02), 2002. pp. 315–322. Paris, France

[4] Wilcox BH, Litwin T, Biesiadecki J, Matthews J, Heverly M, Morrison J, Townsend J, Ahmad N, Sirota A, Cooper B (2007). Athlete: a cargo handling and manipulation robot for the moon. J Field Robot.

[5] Grand C, Ben Amar F, Plumet F, Bidaud P (2004). Stability and traction optimization of a wheel-legged robot. Int J Robot Res.

[6] Endo G, Hirose S. Study on roller-walker (multi-mode steering control and self-contained locomotion). In: Proceedings of the 2000 IEEE international conference on robotics and automation (ICRA’00), 2000, vol 3, pp. 2808–2814. San Francisco, CA, USA

[7] Michaud F, Letourneau D, Arsenault M, Bergeron Y, Cadrin R, Gagnon F, Legault MA, Millette M, Pare JF, Tremblay MC, Lapage P, Morin Y, Bisson J, Caron S. Azimut, a leg-track-wheel robot. In: Proceedings of the 2003 IEEE/RSJ international conference on intelligent robots and systems (IROS’03), 2003, vol 3, pp. 2553–2558. Las Vegas, NV, USA

[8] Murakami H, Sonehara M, Oikawa T, Banno H, Tateishi J (2007). Development of leg-wheeled type mobile robot prototype. IHI Eng Rev.

[9] Dai YJ, Nakano E, Takahashi T, Ookubo H. Motion control of leg-wheel robot for an unexplored outdoor environment. In: Proceedings of the 1996 IEEE/RSJ international conference on intelligent robots and systems (IROS’96), 1996, vol 2, pp. 402–409. Osaka, Japan

[10] Hiller M, Germann D, Morgado de Gois JA. Design and control of a quadruped robot walking in unstructured terrain. In: Proceedings of the 2004 IEEE international conference on control applications (ICCA’04), 2004, vol 2, pp. 916–921. Taipei

[11] Lacagnina M, Muscato G, Sinatra R (2003). Kinematics, dynamics and control of a hybrid robot wheeleg. J Rob Auton Syst.

[12] Hong DW, Jean JB, Ren P. Experimental verification of the walking and turning gaits for a two-actuated spoke wheel robot. In: Proceedings of the 2009 IEEE/RSJ international conference on intelligent robotics and systems (IROS’09), 2009, pp. 402–403. St. Louis, USA

[13] Okada T, Botelho WT, Shimizu T (2010). Motion analysis with experimental verification of the hybrid robot PEOPLER-II for reversible switch between walk and roll on demand. Int J Rob Res.

[14] Steffan E, Das T. Locomotion of circular robots with diametrically translating legs. In: Proceedings of the ASME 2009 dynamic systems and control conference (DSCC’09), 2009, pp. 1–7. Hollywood, CA, USA

[15] Yan J, Agrawal S. Rimless wheel with radially expanding spokes: dynamics, impact, and stable gait. In: Proceedings of the 2004 IEEE international conference on robotics and automation (ICRA’04), 2004, vol 4, pp. 3240–3244. New Orleans, LA, USA

[16] Shores B, Minor M. Design, kinematic analysis, and quasi-steady control of a morphic rolling disk biped climbing robot. In: Proceedings of the 2005 IEEE international conference on robotics and automation (ICRA’05), 2005, pp. 2721–2726. Barcelona, Spain

[17] Shen SY, Li CH, Cheng CC, Lu JC, Wang SF, Lin PC. Design of a leg-wheel hybrid mobile platform. In: Proceedings of the 2009 IEEE/RSJ international conference on intelligent robots and systems (IROS’09), 2009, pp. 4682–4687. St. Louis, USA

[18] Huang KJ, Chen SC, Chou YC, Shen SY, Li CH, Lin PC. Experimental validation of a leg-wheel hybrid mobile robot Quattroped. In: Proceedings of the 2011 IEEE international conference on robotics and automation (ICRA’11), 2011, pp. 2976–2977. Shanghai, China

[19] Consi T, Bingham S, Chepp J, Erdmann T, Mehrotra A, Ringstad J, Zhao B. Amphibious robots as rapidly deployable near-shore observatories. In: Proceedings of the OCEANS 2010, pp. 1–6. Washington DC, USA

[20] Aponick T, Bernstein C. Countermine operations in very shallow water and surf zone: the role of bottom crawlers. In: Proceedings of the OCEANS 2003, pp. 1931–1940. San Diego, CA, USA

[21] Low K, Zhou C, Ong T, Yu J. Modular design and initial gait study of an amphibian robotic turtle. In: Proceedings of the 2007 IEEE international conference on robotics and biomimetics (ROBIO’07), 2007, pp. 535–540. Sanya, China

[22] Wang M, Sun L, Wang Y. Dynamic modelling and optimized energy distribution of amphibian walking robot. In: Proceedings of the 2006 IEEE international conference on mechatronics and automation (ICMA’06), 2006, pp. 634–638. Luoyang, China

[23] Ayers J, Witting J (2007). Biomimetic approaches to the control of underwater walking machines. Philos Trans R Soc A Math Phys Eng Sci.

[24] Crespi A, Badertscher A, Guignard A, Ijspeert A. Swimming and crawling with an amphibious snake robot. In: Proceedings of the 2005 IEEE international conference on robotics and automation (ICRA’05), 2005, pp. 3024–3028. Barcelona, Spain

[25] Crespi A, Ijspeert A. Amphibot ii: an amphibious snake robot that crawls and swims using a central pattern generator. In: Proceedings of the 9th international conference on climbing and walking robots (CLAWAR’06), 2006, pp. 19–27. Brussels, Belgium

[26] Yu S, Ma S, Li B, Wang Y. An amphibious snake-like robot with terrestrial and aquatic gaits. In: Proceedings of the 2011 IEEE international conference on robotics and automation (ICRA’11), 2011, pp. 2960–2961. Shanghai, China

[27] Frejek M, Nokleby S. Design of a small-scale autonomous amphibious vehicle. In: Proceedings of the 2008 Canadian conference on electrical and computer engineering (CCECE’08), 2008, pp. 000781 –000786. Ontario, Canada

[28] Boxerbaum A, Klein M, Bachmann R, Quinn R, Harkins R, Vaidyanathan R. Design of a semi-autonomous hybrid mobility surf-zone robot. In: Proceedings of the 2009 IEEE/ASME international conference on advanced intelligent mechatronics (AIM’09), 2009, pp. 974–979. Singapore

[29] Sun YS, Ma S. ePaddle mechanism: Towards the development of a versatile amphibious locomotion mechanism. In: Proceedings of the 2011 IEEE/RSJ international conference on intelligent robots and systems (IROS’11), 2011, pp. 5035–5040. San Francisco

[30] Sun Y, Ma S (2013). A versatile locomotion mechanism for amphibious robots: eccentric paddle mechanism. Adv Robot.

[31] Sun Y, Ma S, Yang Y (2013). Towards stable and efficient legged race-walking of an ePaddle-based robot. Mechatronics.

[32] Pu H, Zhao J, Sun Y, Ma S, Luo J, Gong Z. Non-reciprocating legged gait for robot with epicyclic-gear-based eccentric paddle mechanism. Robot Auton Syst. 2015;68:36–46. doi:10.1016/j.robot.2015.02.004. http://www.sciencedirect.com/science/article/pii/S09218890150%00226.

[33] Yang Y, Sun Y, Pu H. Paddle trajectory generation for accessing soft terrain by an ePaddle locomotion mechanism. In: Proceedings of the 2013 IEEE international conference on robotics and automation (ICRA’13), 2013, pp. 403–408. Karlsruhe, German

[34] Yang Y, Sun Y, Ma S (2014). Drawbar pull of a wheel with an actively actuated lug on sandy terrain. J Terramech.

[35] Sun Y, Ma S, Fujita K, Yang Y, Pu H. Modeling the rotational paddling of an ePaddle-based amphibious robot. In: Proceedings of the 2012 IEEE/RSJ international conference on intelligent robots and systems (IROS’12), 2012, pp. 610–615. Algarve, Portugal

[36] Pu H, Sun Y, Ma S, Gong Z (2014). Experimental study on the oscillating paddling gait of an epaddle mechanism with flexible configuration. Adv Robot.

[37] Pu H, Sun Y, Yang Y, Ma S. Modeling of the oscillating-paddling gait for an epaddle locomotion mechanism. In: Proceedings of the 2013 IEEE international conference on robotics and automation (ICRA’13), 2013, pp. 3414–3420. Karlsruhe, German

[38] Pu H, Sun Y, Ma S, Gong Z. Experimental study on oscillating paddling gait of an eccentric paddle mechanism. In: Proceedings of the 2012 IEEE international conference on robotics and biomimetics (ROBIO’12), 2012, pp. 187–192. Guanzhou, China (Best Conference Paper Award Finalist).

[39] Ma S, Tomiyama T, Wada H (2005). Omni-directional static walking of a quadruped robot. IEEE Trans Robot.

